# Expression of P2 nucleotide receptors varies with age and sex in murine brain microglia

**DOI:** 10.1186/1742-2094-6-24

**Published:** 2009-08-25

**Authors:** Jessica M Crain, Maria Nikodemova, Jyoti J Watters

**Affiliations:** 1 Department of Comparative Biosciences, University of Wisconsin, Madison, WI 53706, USA; 2 Program in Cellular and Molecular Biology, University of Wisconsin, Madison, WI 53706, USA; 3 Center for Women's Health Research, University of Wisconsin, Madison, WI 53706, USA

## Abstract

Microglia are implicated in multiple neurodegenerative disorders, many of which display sexual dimorphisms and have symptom onsets at different ages. P2 purinergic receptors are critical for regulating various microglial functions, but little is known about how their expression varies with age or sex. Therefore, comprehensive information about purinergic receptor expression in normal microglia, in both sexes, over age is necessary if we are to better understand their roles in the healthy and diseased CNS. We analyzed the expression of all fourteen rodent P2X and P2Y receptors in CD11b^+ ^cells freshly-isolated from the brains of C57Bl/6 mice at five different ages ranging from postnatal day 3 to 12 months, in males and females, using quantitative RT-PCR. We also compared purinergic receptor expression in microglia freshly-isolated from 3 day-old pups to that in primary neonatal microglial cultures created from mice of the same age. We observed patterns in P2 receptor expression with age, most notably increased expression with age and age-restricted expression. There were also several receptors that showed sexually dimorphic expression. Lastly, we noted that *in vitro *culturing of neonatal microglia greatly changed their P2 receptor expression profiles. These data represent the first complete and systematic report of changes in purinergic receptor expression of microglia with age and sex, and provide important information necessary for accurate *in vitro *modeling of healthy animals.

## Introduction

Microglia are the primary resident immune cell population in the central nervous system (CNS). They phagocytose debris following neuronal remodeling processes, help maintain CNS integrity, and perform neuronal support functions through the production of neurotrophins and growth factors [1]. Microglia also react to invading pathogens and CNS damage such as that resulting from physical injury, ischemia, and disease [2,3]. However, uncontrolled microglial activation and their resulting production of neurotoxic cytokines and reactive oxygen and nitrite species is thought to contribute to the pathology of many neurodegenerative disorders. Therefore, agents that function to reduce microglial inflammatory activities are currently being sought.

Work from our laboratory and others' has pointed to a role for P2 purinergic receptors (P2Rs) in reducing microglial production of inflammatory mediators [4-6]. Purines are the endogenous ligands for most P2Rs, but pyrimidines and some nucleotide sugars can activate certain subtypes as well. The two major P2 receptor families are subdivided based on agonist specificities and proposed membrane topologies: the P2X receptors are ligand-gated cation channels composed of homo- or heterotrimeric P2X subunits, and the P2Y receptors are seven transmembrane, G protein-coupled receptors [7]. To date, there are seven known P2X receptor subtypes (P2X1–7) and eight P2Y receptor subtypes (P2Y1, P2Y2, P2Y4, P2Y6, P2Y11, P2Y12, P2Y13, P2Y14) however the P2Y11 receptor gene is absent from the rodent genome [8]. In microglia, nucleotides are important regulators of diverse cellular functions such as release of neuroprotective factors like BDNF [9-12], production of cytokines including TNF-α, IL-1β, and IL-6 [13-15], as well as phagocytic, chemotactic, and motility effects [16,17].

Previous studies have evaluated the microglial expression of specific P2Rs in multiple CNS disease models [18-22], but few have addressed P2 receptor profiles in microglia from healthy animals. Moreover, systematic studies of all fourteen rodent P2Rs in microglia have not been reported. P2 receptors have an important role in modulating microglial inflammatory activity, and this inflammation coincides with the pathology of various neurodegenerative diseases. The diseases described in the studies above (e.g. ALS, Alzheimer's disease) have different incidence rates at different ages; therefore it is essential to know how P2 receptor expression changes with age in the normal CNS as this may have implications for their role in the pathogenesis of disease. In addition, because many CNS diseases are sexually dimorphic (that is, males and females are differentially affected), and microglia are responsive to sex hormones (reviewed in [23]), we were interested in ascertaining if there are differences in microglial P2R expression profiles between males and females.

In the present report, we analyzed P2 receptor expression in brain microglia freshly-isolated from C57Bl/6 mice ranging in age from 3 days to 12 months, and identified several sexual dimorphisms. We also assessed how accurately P2R mRNA levels in mixed sex primary neonatal microglial cultures, commonly used for *in vitro *studies, model expression *in vivo*.

## Materials and methods

### Animals

C57Bl/6 mice were maintained in an AAALAC-accredited animal facility according to protocols approved by the University of Wisconsin Institutional Animal Care and Use Committee. All animals were housed under standard conditions, with a 12 hour light/dark cycle and *ad libitum *food and water. Experiments were performed using animals aged postnatal day 3 (3d), 21 days-old (21d, weaning age), 7 weeks-old (7 wk, adolescent/young adult), 4 months-old (4 mo, adult), and 12 months-old (12 mo, becoming reproductively senescent). Adult male mice were housed in separate cages, while the mature females were housed together for one week prior to use in experiments. The 4 mo females were virgins and the 12 mo mice were retired breeders, with females not having born a litter for at least two months prior to their sacrifice. All efforts were made to minimize the number of animals used yet allow the formation of statistically reliable conclusions.

### CD11b+ cell isolation

Mice were euthanized and then perfused with cold phosphate buffered saline (PBS) to remove the majority of circulating immune cells from the CNS vasculature. The brains (excluding the brain stem but including the cerebellum) were removed, cleaned of meninges, then dissociated into a single cell suspension by both physical disruption and enzymatic digestion using the Neural Tissue Dissociation Kit protocol (this and all associated reagents were from Miltenyi Biotec, Germany unless otherwise stated). Myelin was removed by high-speed centrifugation at 850 g in a 0.9 M solution of sucrose in Hank's Buffered Salt Solution (HBSS; Cellgro, Herndon, VA). Cells were then rinsed in HBSS, resuspended in buffer (PBS, 0.5% BSA, and 2 mM EDTA), and stained with PE-conjugated anti-CD11b antibodies followed by a magnetic bead-conjugated secondary antibody against PE. Magnetically-tagged CD11b^+ ^cells were then isolated using MS columns according to the Miltenyi MACS protocol. Reagents are used at 4° and the cells are kept on ice during the isolation process to slow metabolic rate and reduce the opportunity for microglial cell activation.

**Figure 1:**
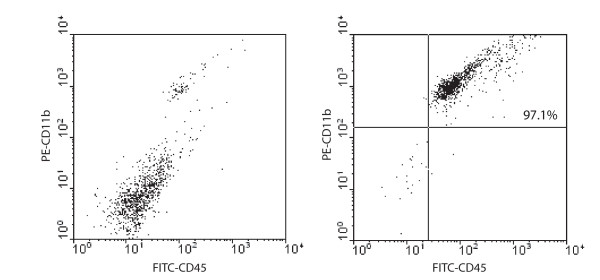
**Purity of freshly-isolated microglia**. CD11b^+ ^cells freshly-isolated from perfused mouse brains as described were co-stained for CD45 and analyzed by flow cytometry. Only a small percentage of mouse brain cells were positive for both markers (left panel). Following magnetic isolation of CD11b^+ ^cells from perfused brains, approximately 97% of the cells were positive for CD11b and CD45 (right panel).

The average purity of isolated cells having the characteristics of microglia was 97% as determined by CD11b/CD45 staining and flow cytometry, including FSC/SSC scatter analysis. Flow cytometric analysis of samples from nonperfused animals demonstrated that the level of CD45 in this population is CD45^low ^whereas blood-derived CD11b^+^ cells were identifiable as a second population that is CD45^high ^(data not shown). These macrophages have been shown to represent only a small fraction of CD45^+ ^cells in the normal CNS of animals perfused with PBS [24]. Similarly, the perfused samples used for this study displayed only a single population of CD45^+ ^cells, as shown in Figure 1, indicating that the cell population isolated by our method contains very few blood-derived cells. As a result, these CD11b^+ ^cells are hereafter referred to as "microglia."

### Genotyping

The sex of the 3d mice was visually determined and later verified by genotyping for the sex-determining region Y (SRY) gene, which is located on the Y chromosome. Genomic DNA was isolated from each mouse by digestion of a small section of tail in a Tris buffer with 0.4 mg/mL proteinase K (Qiagen, Valencia, CA) at 56°, and then used in PCR for SRY using GoTaq Green Master Mix (Promega, Madison, WI). SRY was amplified using the following primers: forward – TCTTAAACTCTGAAGAAGAGAA, reverse – GTCTTGCCTGTATGTGATGG. GAPDH (glyceraldehyde-3-phosphate dehydrogenase) was used as a positive control for DNA quality and successful PCR, and was amplified with the following primers: forward – CCATCACCATCTTCCAGGAG, reverse – GATGGCATGGACTGTGGTC. The PCR cycling program was as follows: 35 cycles of 95° for 2 min; 51° or 60° (for SRY and GAPDH, respectively) for 1 min; and 72° for 40 s, followed by a final extension at 72° for 10 min. The PCR products were electrophoresed on a 1% agarose gel containing ethidium bromide and visualized using the Autochemi Imaging System (UVP, Upland, CA). Visualization of the resulting gels [see Additional file 1] showed that all of the pools were sexed correctly. The consistent detection of GAPDH product demonstrated that DNA was isolated from each pup and could be successfully amplified by PCR even though an SRY amplicon was absent in females.

### Primary microglial cultures

Primary neonatal microglial cultures were prepared as previously described [25]. Litters were approximately 50% female and 50% male. Briefly, 3 day-old C57Bl/6 mice were euthanized, and their brains removed and cleaned of meninges and visible blood vessels. The tissue was dissociated by incubation in 0.25% trypsin (Cellgro) and DNase (Invitrogen Corporation, Carlsbad, CA) followed by trituration. Cells were then pelleted, resuspended in Dulbecco's Modified Eagle's Medium containing 10% fetal bovine serum and penicillin/streptomycin (all from Cellgro), and plated in T75 flasks (Nunc, Rochester, NY) at a density of approximately one brain per flask. The medium was changed the next day, and the cultures were maintained in a humidified incubator with 5% CO_2_. After 10 days, the microglia were lifted from the astrocyte layer by shaking for one hour at 75 rpm on an orbital shaker. The microglia were then plated at 250,000 cells/well in a 12-well plate, cultured for two days, and then harvested. The purity of microglial cultures was greater than 96% as assessed by CD11b^+ ^staining, as described previously [25].

### RNA extraction/reverse transcription

RNA was extracted from all cultured or freshly-isolated microglia according to the TriReagent protocol (Sigma-Aldrich, St. Louis, MO), with the addition of Glycoblue (Ambion, Austin, TX) during the isopropanol incubation. cDNA was synthesized using 1 μg of total RNA and MMLV Reverse Transcriptase (Invitrogen) as previously described [26].

### Quantitative PCR

cDNA was used in real-time quantitative PCR with Power SYBR Green (Applied Biosystems) using the ABI 7300 system. The PCR cycling program was as follows: 95° for 15 min, followed by 45 cycles of 95° for 15 s and 60° for 1 min, and finally a dissociation stage. The primer sequences are as follows, and were designed to span introns whenever possible to discount any product from genomic DNA. Primer specificity was assessed through NCBI BLAST analysis prior to use and, for each sample following PCR, verification that the dissociation curve had a single peak with an observed T_m _consistent with the amplicon length. Primer efficiency was tested through the use of serial dilutions.

Ct values from duplicate measurements were averaged, and relative expression levels were determined by the ΔΔCt method. Evaluation of at least four different housekeeping genes, with multiple primer sets, indicated that the expression levels of β-actin and β-tubulin remained most stable in the microglia over age. Therefore, we chose to normalize the expression of each gene to the levels of β-actin detected in the same sample using the following primer sets: F – ACCCTAAGGCCAACCGTGAA, R – AGAGCATAGCCCTCGTAGATGG; or: F – CACAGCTTCTTTGCAGCTCCTT, R – ACGACCAGCGCAGCGATAT. The average Ct for β-actin was 18; the average Ct values for the P2Rs (at 3d) are included in the primer sequence table (Table 1) as the number of cycles to threshold above that for β-actin. mRNA levels for some genes, including the absence of P2X2, were verified using a second unique primer set. While PCRs were run to 45 cycles, all detected genes had Ct values below 35 in a majority of the samples examined.

**Table 1:** Primer sequence table

Gene	Forward primer	Reverse primer	Average Ct (vs. β-actin) for 3d samples
P2X1	CAGAAAGGAAAGCCCAAGGTATT	CACGTCTTCACAGTGCCATTG	+9

P2X2	GCTGCTCATTCTGCTTTACTTCG	TCCCACACTTTGTGTTCCGA	N.A.

P2X3	AAGGCTTCGGACGCTATGC	GATGACAAAGACAGAAGTGCCCT	+16

P2X4	AGACGGACCAGTGATGCCTAAC	TGGAGTGGAGACCGAGTGAGA	+6

P2X5	GATGTGGCAGACTTTGTCATTCC	CCTTCACGCTCAGCACAGATG	+4

P2X6	ACGTGTTCTTCCTGGTAACCAACT	TGGACATCTGCCCTGGACTT	+13

			

P2X7	ACAATGTGGAAAAGCGGACG	TCAATGCACACAGTGGCCA	+4

P2Y1	AGCAGAATGGAGACACGAGTTTG	GGGATGTCTTGTGACCATGTTACA	+16

P2Y2	GAAGAACTGGAGCAGGCGCT	CCATTGCCCTGGACCTGATC	+12

P2Y4	CTGCAAGTTCGTCCGCTTTC	GTATTGCCCGCAGTGGATG	+16

P2Y6	TGAAAACAACGAGGAACACCAA	CAGCCTTTCCTATGCTCGGA	+6

P2Y12	CACAGAGGGCTTTGGGAACTTA	TGGTCCTGCTTCTGCTGAATC	+3

P2Y13	CAGCTGAGTCTCTTCCAAAACAAA	TGCATCCCAGTGGTGTTGAT	+5

P2Y14	CCACCACAGACCCTCCAAAC	CAACACGGGAATGATCTGCTTT	+14

### Statistical analysis

**Figure 2:**
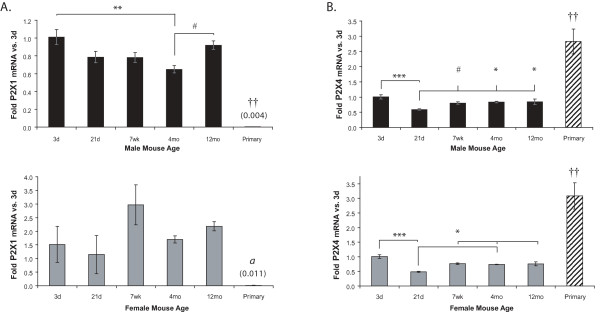
**P2X1 (A) and P2X4 (B) receptor mRNA expression in freshly-isolated mouse microglia varies with age and differs from cultured neonatal microglia**. P2R mRNA levels in freshly-isolated microglia from male and female mice of different ages and mixed-sex primary neonatal microglial cultures were determined using qRT-PCR. Expression was normalized to β-actin and average folds (+/- SEM) are graphed relative to the 3d time point of each sex. n = 3–6; for primary cells n = 5 separate culture preparations. Values in parentheses are fold-changes of the lowly-expressed samples. ^# ^0.05 < p ≤ 0.10, * p ≤ 0.05; ** p ≤ 0.01; *** p ≤ 0.005. Primary cultures: ^†† ^p ≤ 0.01 vs. all freshly-isolated microglia, *a *- p ≤ 0.01 vs. 7 wk and 12 mo.

**Figure 3:**
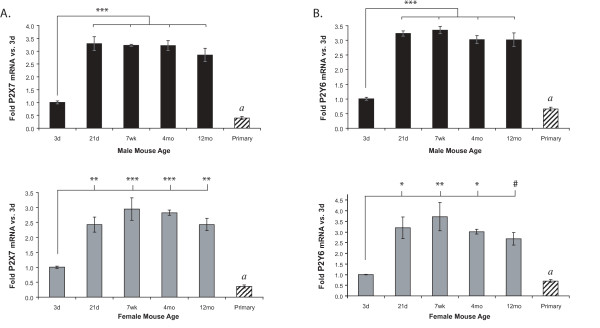
**P2X7 (A) and P2Y6 (B) mRNA expression in microglia is lower at 3 days than at the other ages**. P2R mRNA levels in freshly-isolated microglia from male and female mice of different ages and mixed-sex primary neonatal microglial cultures were determined using qRT-PCR. Expression was normalized to β-actin and average folds (+/- SEM) are graphed relative to the 3d time point of each sex. n = 4–6; for primary cells n = 5 separate culture preparations. ^#^0.05 < p ≤ 0.10; * p ≤ 0.05; ** p ≤ 0.01; *** p ≤ 0.005. Primary cultures: *a *- 0.05 < p ≤ 0.1 vs. freshly-isolated microglia.

**Figure 4:**
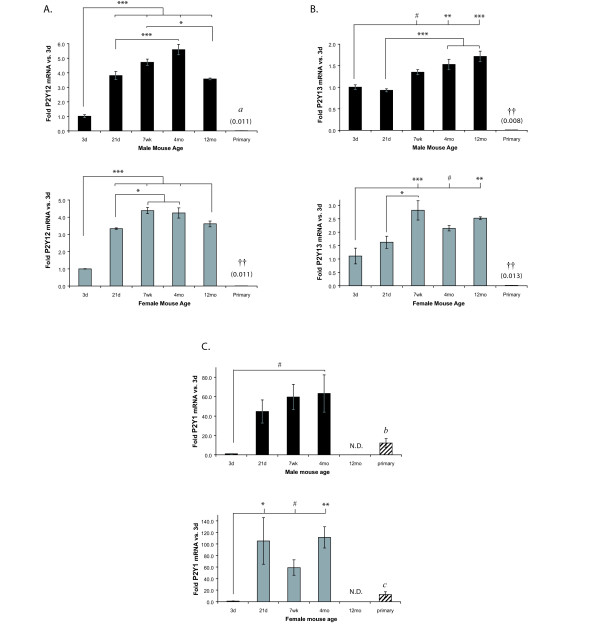
**P2Y12 (A), P2Y13 (B), and P2Y1 (C) mRNA expression increases with age**. P2R mRNA levels in freshly-isolated microglia from male and female mice of different ages and mixed-sex primary neonatal microglial cultures were determined using qRT-PCR. Expression was normalized to β-actin and average folds (+/- SEM) are graphed relative to the 3d time point of each sex. n = 3–6; for primary cells n = 5 separate culture preparations. Values in parentheses are fold-changes of the lowly-expressed samples. ^# ^0.05 < p ≤ 0.10; * p ≤ 0.05; ** p ≤ 0.01; *** p ≤ 0.005. Primary cultures: ^†† ^p ≤ 0.01 vs. all freshly-isolated microglia; *a *- 0.05 < p ≤ 0.1 vs. 21 d, 7 wk, 4 mo, 12 mo and p ≤ 0.05 vs. 3 d; *b *- p ≤ 0.05 vs. 7 wk and 4 mo; *c *- p ≤ 0.01 vs. 21 d and 4 mo. N.D. = not detected.

**Figure 5:**
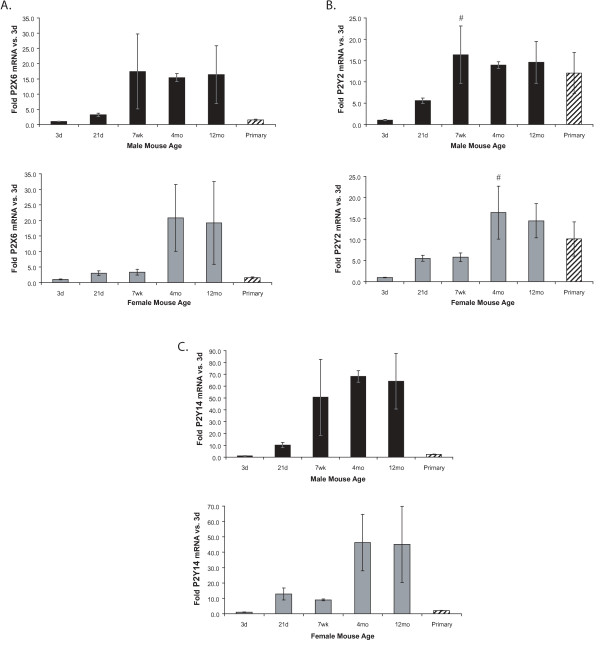
**P2X6 (A), P2Y2 (B), and P2Y14 (C) mRNA expression does not significantly change with age**. P2R mRNA levels in freshly-isolated microglia from male and female mice of different ages and mixed-sex primary neonatal microglial cultures were determined using qRT-PCR. Expression was normalized to β-actin and average folds (+/- SEM) are graphed relative to the 3d time point of each sex. n = 4–6; for primary cells n = 5 separate culture preparations. ^# ^0.05 < p ≤ 0.10 vs. 3d.

**Figure 6:**
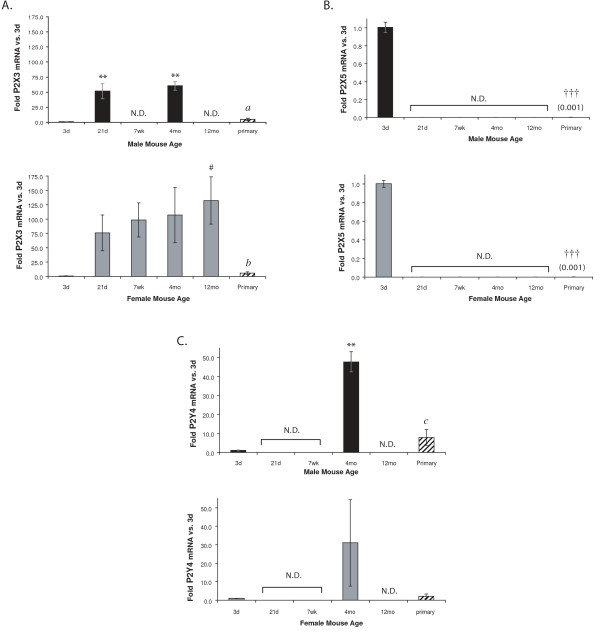
**P2X3 (A), P2X5 (B), and P2Y4 (C) mRNA is expression is not detectable in microglia freshly-isolated from some ages of mice**. P2R mRNA levels in freshly-isolated microglia from male and female mice of different ages and mixed-sex primary neonatal microglial cultures were determined using qRT-PCR. Expression was normalized to β-actin and average folds (+/- SEM) are graphed relative to the 3d time point of each sex. n = 3–5; for primary cells n = 4–5 separate culture preparations. Values in parentheses are fold-changes of the lowly-expressed samples. ^# ^0.05 < p ≤ 0.10; ** p ≤ 0.01; *** p ≤ 0.005. Primary cultures: ^††† ^p ≤ 0.005 vs. all freshly-isolated microglia; *a *- p ≤ 0.05 vs. 21 d and 4 mo; *b *- p ≤ 0.05 vs. 12 mo; *c *- p ≤ 0.01 vs. 4 mo. N.D. = not detected.

Statistical analyses were performed on ΔΔCt data using a one-way ANOVA followed by the Tukey-Kramer Multiple Comparisons or Dunnett *post hoc *tests (as appropriate) or unpaired t-tests with a Welch correction, using Sigma Stat 3.1 software. Statistical significance was set at the 95% confidence limit (p < 0.05). For Figures 2, 3, 4, 5 and 6, a single symbol (* or †) above a bar represents p < 0.05; two symbols p < 0.01; three symbols p < 0.001; and a letter or # above a bar represents 0.05 < p < 0.10. Quantitative data are expressed as the mean ± SEM of n = 4–6 mice in each group (males in black bars, females in grey). Due to the limited amount of tissue, brains from three or four 3d mice were pooled by sex [see Additional file 1], and microglia were isolated from n = 4 independent pools for each sex. Results for primary microglial cultures (hatched bars) are from n = 5 independent culture preparations. Levels of gene expression are shown relative to 3 day-old animals (for age comparisons) or males (for male/female comparisons).

## Results

In the present study, we evaluated the expression of all rodent P2Rs in freshly-isolated mouse microglia, and we display them here in groups according to their overall expression patterns. We found four major expression patterns: expression that varies over age (Figure 2); expression that increases with age (Figures 3 and 4); expression that does not change with age (Figure 5); and age-restricted expression (Figure 6).

### P2X1 and P2X4 expression varies over age

We found that mRNA expression of both P2X1 (Figure 2A) and P2X4 (Figure 2B) vary with age. In males (Figure 2A, upper panel), P2X1 levels are significantly lower at 4 mo than they are at 3d, but no statistically significant differences were found in females (Figure 2A, lower panel). In contrast, P2X4 expression is lower at 21d than at the other ages (Figure 2B). Surprisingly, levels of both receptors in primary cultures are very different compared to those in freshly-isolated microglia from mice at any age examined, with P2X1 being significantly lower and P2X4 expression being approximately three times more abundant.

### Expression of P2X7 and P2Y6 is steady after 21d

P2X7 (Figure 3A) and P2Y6 (Figure 3B) mRNA levels are lowest at 3d in both males and females, and were approximately three-fold higher at all other ages. Thus, maximum expression of both of these receptors appears to be on or before 21 days of age, a time coinciding with weaning. In addition, mRNA levels of both receptors in primary cultures are similar to those in microglia isolated from 3d pups.

### Expression of P2Y12, P2Y13, and P2Y1 increases with age

P2Y12 (Figure 4A), P2Y13 (Figure 4B), and P2Y1 (Figure 4C) mRNA levels in freshly-isolated microglia were also lowest at 3d, but they continued to increase significantly with age. In both males and females, P2Y12 expression was lower at 3d than at any other age. Its expression also appeared to peak in both sexes by 4 mo (by ~five-fold), but in males it decreased significantly thereafter (Figure 4A, upper panel). In addition, P2Y12 expression was significantly lower (by ~100-fold) in primary microglial cultures than in any of the freshly-isolated cells.

Like P2Y12 mRNA levels, P2Y13 expression is also much lower (~100-fold) in primary cultures than in the freshly-isolated microglia (Figure 4B). Expression is increased in microglia from 7 wk, 4 mo, and 12 mo animals compared to 3d animals, in both sexes, though to varying levels of significance. In males, P2Y13 levels were also significantly higher than in the 21d samples, but in females only, the 7 wk time point was also higher than 21d.

P2Y1 expression in 21 d, 7 wk, and 4 mo animals also trended towards increased expression (by 50- to 100-fold) compared to 3 d old animals (Figure 4C); but unlike like P2Y12 and P2Y13, its expression in 3d microglia and primary microglial cultures was similar. Unexpectedly, we found that P2Y1 receptor expression was not detectable in 12 mo microglia samples.

### Increases in P2X6, P2Y2, and P2Y14 with age are not significant

In contrast to the receptors described above, the levels of P2X6 (Figure 5A), P2Y2 (Figure 5B), and P2Y14 (Figure 5C) did not significantly change with age, although they all showed a trend toward increased levels of expression at adult ages (4 mo and 12 mo). The trend would likely become significant if a larger number of animals was used, though this sample size produced highly significant results for other, less variable P2 receptors.

### Expression of P2X3, P2X5, and P2Y4 is age-restricted

We found an unusual pattern of expression for P2X3 (Figure 6A), P2X5 (Figure 6B), and P2Y4 (Figure 6C) receptors: their expression in microglia appeared to be restricted to mice of specific ages. For example, P2X5 is present in microglia from 3d mice, but is absent in microglia from males and females of all the other examined ages (Figure 6B). P2X5 expression in primary neonatal microglial cultures is significantly lower (by ~800-fold) than in the freshly-isolated 3d microglial samples. P2Y4 mRNA is expressed in microglia from 3d and 4 mo mice, but it was not detected in samples from other mouse ages (Figure 6C). Its expression in 4 mo male samples is significantly higher than in 3d or primary microglia, but in 4 mo females this difference did not reach statistical significance. Lastly, in females, P2X3 mRNA was low but detectable at 3d, and it increased with age from 21d to 12 mo (by ~75-fold to 125-fold). However, while P2X3 mRNA is also increased in males at 21d (~50-fold) and 4 mo (~60-fold) compared to 3d, it was undetectable in microglia from 7 wk or 12 mo animals (Figure 6A). P2X3 mRNA levels in primary microglia were similar to microglia isolated from 3d mice of both sexes.

P2X2 mRNA was not detected in freshly-isolated microglia from male or female mice at any age examined, nor was it detected in our neonatal primary microglial cultures. These observations were confirmed using at least two P2X2 primer sets which did amplify P2X2 from whole brain cDNA samples.

### Expression of some P2 receptors is sexually dimorphic

**Figure 7:**
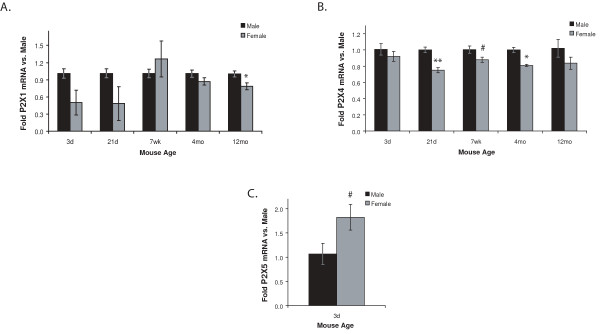
**P2X1 (A), P2X4 (B), and P2X5 (C) mRNA expression is sexually dimorphic**. P2R mRNA levels in freshly-isolated microglia from male and female mice of different ages were determined using qRT-PCR. Expression was normalized to β-actin and average folds (+/- SEM) are graphed relative to the males at each age. n = 3–6. ^# ^0.05 < p ≤ 0.10; * p ≤ 0.05.

In order to examine the effect of sex on P2R expression, independently of age effects, we compared expression in freshly-isolated microglia from females to that in males at each age. For the P2X receptors, we found sexual dimorphisms in four members: 1) P2X1 expression (Figure 7A) is significantly decreased in 12 mo females compared to age-matched males; 2) expression of P2X4 (Figure 7B) is significantly lower in microglia from females than males at 21 d, 7 wk, and 4 mo; 3) P2X5 levels (Figure 7C) in females are nearly twice those in males at 3d (the only age at which it was detected) and 4) the P2X3 receptor is expressed in microglia isolated from females at 7 wk and 12 mo, but it is not detected in microglia from male mice of these ages (Figure 6A).

**Figure 8:**
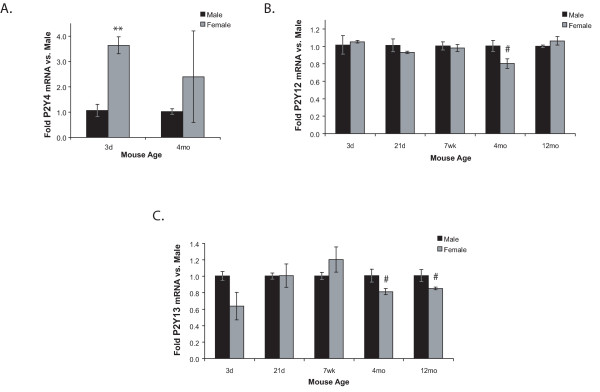
**P2Y4 (A), P2Y12 (B), and P2Y13 (C) mRNA expression is sexually dimorphic**. P2R mRNA levels in freshly-isolated microglia from male and female mice of different ages were determined using qRT-PCR. Expression was normalized to β-actin and average folds (+/- SEM) are graphed relative to the males at each age. n = 3–6. ^# ^0.05 < p ≤ 0.10; ** p ≤ 0.01.

We also found sexual dimorphisms in three P2Y receptors: 1) P2Y4 mRNA levels (Figure 8A) are nearly four times higher in freshly-isolated microglia from 3d females than from males, and there is a trend, though not significant, at 4 mo as well; 2) P2Y12 expression (Figure 8B) is lower at 4 mo in female microglia than that in males; and 3) P2Y13 expression is lower at 4 mo and 12 mo in female microglia (Figure 8C).

No statistically significant sexual dimorphisms were found in the other six P2 receptors.

### In vitro culturing of microglia alters their P2 receptor expression

Primary cultures derived from early postnatal animals are commonly used to study microglial P2 nucleotide receptor function; therefore it is important to ascertain how accurately these cultures represent microglia *in vivo*. We compared P2R expression in mixed-sex primary microglial cultures (made from 3 day-old pups) to the average of that in freshly-isolated microglia from 3d males and females, and found differences in 10 of the 13 expressed receptors (Table 2). P2X1, P2X5, and P2X7 mRNA levels were significantly decreased, whereas P2X4 levels were increased in primary cultures. Of particular note is the dramatic reduction in P2X1 and P2X5 mRNA levels (by more than 150-fold) in culture relative to freshly-isolated microglia. Among the P2Y receptors, all but P2Y4 showed a significant, or nearly significant, difference in expression in primary versus freshly-isolated microglia. P2Y1, P2Y2, and P2Y14 were increased in cultured microglia whereas P2Y6, P2Y12, and P2Y13 mRNA levels were significantly decreased in primary cultures compared to those in freshly-isolated microglia from mice of the same age, with P2Y12 and P2Y13 levels being reduced by nearly 100-fold.

**Table 2:** In vitro culturing of microglia alters their P2 receptor expression profiles.

Gene	Fold 1° vs. 3d
	Ave. of ♂ and ♀ (+/- SEM)
P2X1	↓167 (+/- 83) ***

P2X3	↑ 5.39 (+/- 2.07)

P2X4	↑ 2.95 (+/- 0.43) *

P2X5	↓ 833 (+/- 347) ***

P2X6	↑ 1.52 (+/- 0.32)

P2X7	↓ 2.65 (+/- 0.38) ***

P2Y1	↑ 12.3 (+/- 4.61) ^#^

P2Y2	↑ 11.1 (+/- 4.41) ^#^

P2Y4	↑ 4.54 (+/- 2.41)

P2Y6	↓ 1.47 (+/- 0.14) **

P2Y12	↓ 90.9 (+/- 16.5) ***

P2Y13	↓ 100 (+/- 20.0) ***

P2Y14	↑ 2.22 (+/- 0.20) ***

P2R mRNA levels in freshly-isolated microglia from 3d male and female mice and mixed-sex primary neonatal microglial cultures (also derived from 3d mice) was determined using qRT-PCR. Expression was normalized to β-actin and the average fold change (+/- SEM) of the mixed primary cultures is given compared to the average of male and female 3d freshly-isolated microglia. n = 3–4 for freshly-isolated samples; n = 4–5 separate culture preparations for primary cells. # p < 0.10, * p < 0.05, ** p < 0.01, *** p < 0.001.

## Discussion

In the present studies, we comprehensively analyzed P2X and P2Y purinergic receptor expression levels in freshly-isolated brain microglia from male and female mice ranging in age from 3 days to 12 months. No studies to date have reported changes in the basal expression of all P2 receptors in microglia with animal age. Moreover, sexual dimorphisms in P2R expression in microglia have not been studied before, despite strong gender differences in multiple neurodegenerative diseases in which microglial inflammatory activities are implicated. Lastly, we assessed how P2R expression is altered by culturing *in vitro*, a significant issue given that primary mixed-sex neonatal microglia are widely used for microglial studies.

The cells used in this study were CD11b^+ ^cells isolated from PBS-perfused C57Bl/6 mouse brain. The C57Bl/6 strain was chosen because it is a common research strain and it is the genetic background upon which many knockout mice are made. Of the isolated CD11b^+ ^cells used in this study, an average of 97% were also CD45^+^. Because the majority of circulating CD11b^+ ^cells were removed by PBS perfusion prior to brain harvesting, the contribution of non-microglial cells was negligible, consistent with previous reports [24]. The magnetic bead isolation system we employed for isolating CD11b^+ ^cells provided enough cells to allow for quantitative analysis by RT-PCR without the need for culturing. Moreover, this system enabled the rapid isolation of microglial cells from multiple samples simultaneously and minimized sample degradation, compared to cell sorting by standard flow cytometry.

The majority of genes examined in this study showed relatively minimal variability for *ex vivo *samples. A few of the more lowly-expressed genes (P2X3, P2X6, P2Y2, P2Y14) displayed higher variation. This probably reflects a wider range of expression levels for these genes within the population, but it may also be due to the influence of gonadal hormones as discussed further below, or the result of short-term effects of mouse activity (e.g. differences between when each mouse last ate, exercised, etc.) prior to harvest. The absence of detection of P2X3 in males at 7 wk and 12 mo, and P2Y4 in both sexes at all ages examined except 3d and 4 mo is likely to be specific to the expression of those particular genes. For example, the expression of many receptors is indeed at their maximum at the 7 wk time point and several internal controls for the integrity of each sample were used in this study (including the assessment of housekeeping genes measured using multiple primer sets), suggesting that sample quality is not the reason for the absence of expression of one or two particular genes out of fifteen.

### Age-dependent changes in P2X receptors

Whereas there have been descriptions of P2R expression in a number of organ systems (reviewed in [27]), to our knowledge this is the first study examining both P2X and P2Y expression in immune cells at different ages. Several studies have evaluated changes in specific P2X subtypes in various CNS disease models [18-22] and found their expression to be increased. Given that nucleotides can modulate cytokine levels [13-15] and inflammation is often present in these diseases, P2Rs may have a role in the development or initiation of these conditions. Here we report that microglial P2X receptor expression varies with mouse age, although with differing time courses.

While little is known concerning the specific inflammatory roles of most P2X receptors in microglia, P2X4 and P2X7 receptor function is better understood. P2X4 up-regulation has an identified role in neuropathic pain [28], and P2X7 receptors are critical for controlling inflammasome assembly that leads to cytokine release [29-31]. We found that P2X4 expression was decreased in microglia from 21d mice compared to that from the other ages, while P2X7 levels were lowest at 3d and approximately three-fold higher in microglia from mice at the other ages examined. It is interesting to note that a recent report indicates that increased P2X7 levels drive microglial activation and proliferation *in vitro *[32], an effect that may predispose to Neuroinflammation in adults. A previous study of P2X receptor expression in late embryonic and early postnatal rat brain [33] found that P2X1 immunoreactivity declined to undetectable levels at postnatal days 30 and 60 (males and females were not distinguished). Although those time points do not match our own, we found relatively steady expression of P2X1 mRNA in microglia from mice even to 12 months of age in both sexes. This discrepancy may be due to differences in assay sensitivity between qRT-PCR and immunohistochemical staining methods, or simple species differences. Differences between microglial markers used may also account for this divergence. For example, Xiang and Burnstock saw dissimilar results for certain P2X receptors when ED-1 or isolectin B4 was used as the microglial co-stain; we used CD11b as the microglial marker in our studies. It is also possible that there are disparities between P2X1 mRNA and translated protein levels, however, post-transcriptional regulation of P2X1 receptors has not been documented.

P2X2 mRNA was not detected in any of the samples examined, and P2X5 receptor mRNA was only detectable in microglia from 3d-old animals. Similar to our results, Xiang and Burnstock did not detect P2X2 immunoreactivity in microglia from animals examined up to 60 days of age. However, they did observe transient P2X5 receptor immunoreactivity in cerebellar microglia in 7 day-old rats [34], a time point we did not examine.

Lastly, this is the first study demonstrating expression of P2X3 and P2X6 mRNA in freshly-isolated mouse brain microglia, even though these receptors have been reported in the murine N9 microglial cell line [35]. This pattern of P2X6 receptor expression steadily increasing with age until it plateaus at about 7 wk of age is consistent with P2X6 expression in rat Kupffer cells where it also increases with age [36].

### Age-dependent changes in P2Y receptors

Although much less is known about the function of P2Y receptors in microglia compared to P2X receptors, the importance of P2Y receptors in regulating various microglial activities is becoming clear [37]. Recently, critical roles for P2Y6 receptors in microglial phagocytosis [38] and P2Y12 receptors in microglial migration, chemotaxis and process extension have been revealed [16,17,39,40]. In addition, a role for P2Y12 receptors in neuropathic pain has also recently been described [41].

In the present study, we found that the expression of most P2Y receptors tended to increase between postnatal days 3 and 21, except for that of P2Y13 and P2Y4. The expression of P2Y1, P2Y6 and P2Y12 receptors seemed to be most strongly up-regulated during this time. There is significant synaptic re-modeling and pruning that occurs during this period in mouse brain development [42,43], and microglial activities are essential for these processes [44-46]. While the specific roles of these receptors in microglia early in postnatal life is not yet known, we speculate given the central role of P2Y6 receptors in microglial phagocytosis [39] and P2Y12 receptors in chemotaxis and migration, that their up-regulation may facilitate these activities during brain development.

Also important, P2Y12 receptor expression levels declined between 4 months and 12 months of age. Similarly, P2Y1 and P2Y4 receptor levels are completely undetectable in microglia from 12 mo animals. A decline in P2 receptors whose expression is requisite for microglial migration and phagocytosis may contribute to microglial senescence, a hypothesis proposing that microglia lose their normal capacity to perform neuron-protective and -supportive functions as they age [45,46]. Another interesting point is that the phylogenetically and structurally related P2Y receptors (i.e. P2Y1, P2Y2, P2Y4, and P2Y6) which are postulated to inhibit inflammatory mediator release from microglia [4-6], have either decreasing expression with age or are completely undetectable in 12 mo animals. If the endogenous role of these P2Y receptors in microglia is indeed to act as a natural "brake" for pro-inflammatory mediator production, perhaps their decline, as in Alzheimer's disease [18-22], underlies the exaggerated microglial inflammatory responses of the aged brain, and may contribute to the onset of inflammatory processes involved in neurodegenerative disease pathology [45,46]. Studies are in progress using RNAi methods similar to those used in [47] to investigate the effect of receptor knockdown on pro-inflammatory cytokine expression in aged brains.

### Sexual dimorphisms

Seven P2 receptors were found to be sexually dimorphic in freshly-isolated microglia, though the ages at which these differences occurred varied depending on the receptor. Sexual dimorphisms were found in P2X1, P2X4, P2Y12, and P2Y13 expression which was lower in females than males, whereas P2X3, P2X5, and P2Y4 mRNA levels were higher in microglia from females compared to age-matched males.

At both ages when P2Y4 was expressed (3 d and 4 mo), microglia from females had higher expression than those from males. Although this difference was only statistically significant at 3d, the high variability in P2Y4 expression in females at 4 mo may result from hormonal regulation of this gene. While breeding-age female mice in this study were housed together, we did not identify estrous stage prior to their sacrifice. Gonadal steroid hormone regulation of the P2Y4 gene has not been described, but we have identified several putative estrogen response elements in its 5' flanking region (JMC and JJW unpublished observations). Future studies will investigate the effect of estrous cycle stage on P2Y4 expression in microglia. It should also be noted here that the P2Y4 gene is located on the X chromosome in the mouse, human and rat, and it is the only known P2R encoded on this chromosome.

In males, we find P2X3 mRNA levels in microglia to be undetectable in 7 wk and 12 mo animals, whereas its expression in females gradually increased between 21d and 12 months. In addition, because we find that P2X3 mRNA levels decline dramatically between day 21 and 7 wks in males, there may be a role for maternal hormones in regulating its expression in early postnatal male pups. There is some evidence supporting P2X3 receptor regulation by gonadal hormones in peripheral tissues: ovariectomy has been shown to alter P2X3 expression in mouse bladder [48], and its expression is increased in the cervix during pregnancy [49]. We also observed that P2X1 mRNA levels were significantly lower in microglia from 12 mo females. While the expression of all P2X receptor subtypes has been evaluated in the adult female rat reproductive tract, only a single estrous stage was examined [50], and therefore possible changes in P2X receptor expression patterns due to alterations in circulating steroid hormone levels could not be addressed. We are currently completing studies that more specifically evaluate the role of female sex hormones in regulating P2R expression in microglia.

### Differences between freshly-isolated microglia and primary cultures

This study demonstrates that culturing neonatal microglia greatly alters their P2R expression when compared to microglia freshly-isolated from 3d mice, the age used for making the primary cultures. P2 mRNA levels in averaged 3d male and female freshly-isolated microglia were found to differ from that in primary mixed-sex microglial cultures by three- to over 800-fold. Culturing conditions can alter microglial gene expression in a number of ways, including their exposure to soluble factors in serum at concentrations different from those in the CNS, the absence of neurons and synaptic connections in a dish, as well as altered microglia:astrocyte ratios from which they are exposed *in vivo*. Thus, it is not unexpected that microglia, a cell type whose primary function is to monitor their surroundings, change in response to alterations in their environment. However, to our knowledge this is the first report that begins to address how well primary neonatal microglial cultures model expression in mice of different ages and demonstrates that culturing significantly alters P2R levels in microglia.

We also compared P2 receptor profiles in primary microglia to each of the other ages of freshly-isolated microglia. While there were instances where expression in primary microglia was similar to that in freshly-isolated cells, overall there was not one particular age that was most accurately represented by the P2R expression in primary cultures. As with most *in vitro *experiments, extrapolation of results obtained using primary microglia as a model (and microglial cell lines as well), should therefore be interpreted cautiously. However primary cultures do, at a minimum, provide a good model for studying P2X6, P2X7, and P2Y6 signaling in 3d mice, or P2Y2 signaling in adult mice. Additionally P2Y1, P2Y14, and P2X3 expression in primary cultures is in between the expression levels of microglia from 3d and 21 day-old mice.

### Conclusions and future directions

The data presented here provide new, sensitive and quantitative information on the impact of age and sex on P2R mRNA expression in microglia. Although evaluating P2R protein levels is desirable, P2X receptor mRNA levels closely correlate with protein levels [51-53], and additional information provided by such studies would be limited. Accurate quantitative analysis is difficult with immunohistochemical staining, and immunoblot analysis would not allow discrimination between cell surface receptors and intracellular pools. Several P2X immunohistochemical studies have been performed in rats by Burnstock and colleagues (some using custom-made antibodies), however, the commercially-available antibodies for mouse P2Rs (including many we have evaluated for P2YRs) often show non-specific bands in immunoblots which would be indistinguishable in immunohistochemistry ([54]; JJW unpublished observations). Therefore, future development of antibodies having greater target specificity is needed to provide more reliable information on regulation of P2X and P2Y receptors at the protein level. Additionally, as P2X channel activity is dependent on subunit composition, FRET labeling studies using highly-specific antibodies to identify individual P2X subunit constituents of native channels in microglia would be very informative.

In conclusion, the present studies provide the most comprehensive information to date on the changes in P2 receptor expression in freshly-isolated microglia with age and represent the first such study of P2YRs. These data contribute to a better understanding of these receptors, which have important effects on a host of microglial activities, in the normal aging CNS. Microglial inflammatory activities in particular are implicated in the pathology of a number of neurodegenerative diseases which emerge at different ages. Many of these conditions are strongly sexually dimorphic, and we identified several sexual dimorphisms in P2 receptor expression. In addition, we found many differences in P2R levels between primary neonatal microglia and freshly-isolated microglia, which begins to address the usefulness and accuracy of this current gold standard for modeling purinergic signaling *in vitro*. Together, these data provide important new information necessary for future mechanistic studies on the contribution of P2Rs to the aging process and their role in CNS inflammatory disease.

## Competing interests

The authors declare that they have no competing interests.

## Authors' contributions

JC carried out all animal experiments, RNA analyses, generated the figures and performed statistical analyses. MN performed the tissue culture experiments and provided samples for RNA analyses. JW designed the study and obtained funding. JC and JW drafted the manuscript and JW helped with statistical analyses. All authors read and approved the final manuscript.

## Supplementary Material

Additional file 1 **Sex verification of pooled 3d mice**. Tail samples from individual mice in each pool (three to four mice/pool) were used in genotyping for SRY. Each lane is the product from an individual mouse. GAPDH is used as a positive control for PCR. ♀-Sample from adult female mouse ear, serving as a negative control for SRY genotyping. ♂-Sample from adult male mouse ear, serving as a positive control for SRY genotyping. Click here for file
