# Supplementary figures and images for: Expression of P2 nucleotide receptors varies with age and sex in murine brain microglia
Source: J Neuroinflammation. 2009 Aug 25;6:24. doi: 10.1186/1742-2094-6-24 (PMC2744668; doi:10.1186/1742-2094-6-24)

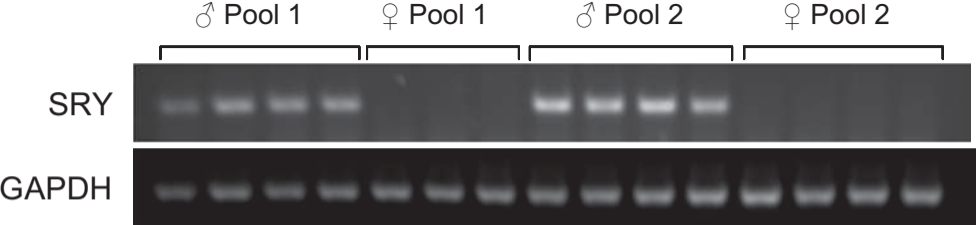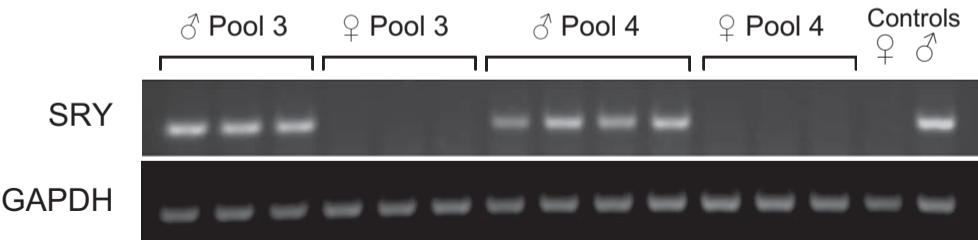

Supplement: Additional file 1 — Sex verification of pooled 3d mice. Tail samples from individual mice in each pool (three to four mice/pool) were used in genotyping for SRY. Each lane is the product from an individual mouse. GAPDH is used as a positive control for PCR. ♀-Sample from adult female mouse ear, serving as a negative control for SRY genotyping. ♂-Sample from adult male mouse ear, serving as a positive control for SRY genotyping. [file 1742-2094-6-24-S1.pdf]
